# Perpendicular magnetic tunnel junctions with a synthetic storage or reference layer: A new route towards Pt- and Pd-free junctions

**DOI:** 10.1038/srep21246

**Published:** 2016-02-17

**Authors:** Léa Cuchet, Bernard Rodmacq, Stéphane Auffret, Ricardo C. Sousa, Ioan L. Prejbeanu, Bernard Dieny

**Affiliations:** 1 Univ. Grenoble Alpes, INAC-SPINTEC, F-38000 Grenoble, France; 2 CEA, INAC-SPINTEC, F-38000 Grenoble, France; 3 CNRS, SPINTEC, F-38000, Grenoble, France

## Abstract

We report here the development of Pt and Pd-free perpendicular magnetic tunnel junctions (p-MTJ) for STT-MRAM applications. We start by studying a p-MTJ consisting of a bottom synthetic Co/Pt reference layer and a synthetic FeCoB/Ru/FeCoB storage layer covered with an MgO layer. We first investigate the evolution of RKKY coupling with Ru spacer thickness in such a storage layer. The coupling becomes antiferromagnetic above 0.5 nm and its strength decreases monotonously with increasing Ru thickness. This contrasts with the behavior of Co-based systems for which a maximum in interlayer coupling is generally observed around 0.8 nm. A thin Ta insertion below the Ru spacer considerably decreases the coupling energy, without basically changing its variation with Ru thickness. After optimization of the non-magnetic and magnetic layer thicknesses, it appears that such a FeCoB/Ru/FeCoB synthetic storage layer sandwiched between MgO barriers can be made stable enough to actually be used as hard reference layer in single or double magnetic tunnel junctions, the storage layer being now a single soft FeCoB layer. Finally, we realize Pt- or Pd-free robust perpendicular magnetic tunnel junctions, still keeping the advantage of a synthetic reference layer in terms of reduction of stray fields at small pillar sizes.

The field of memory applications is becoming more and more challenging as the performances required by the microelectronic industry increase. In that context, Magnetic Random Access Memories (MRAM) appear as competitive candidates that provide non-volatility, high storage densities, high write speed of a few ns, possibly infinite endurance (>10^16^ write cycles) and radiation hardness.

The discovery of very large TMR amplitude in in-plane magnetized magnetic tunnel junctions (MTJ) with a crystalline MgO barrier has been a major breakthrough^1^,^2^. However, for memory applications, the interest rapidly evolved towards out-of-plane magnetized systems. Indeed, using MTJs with Perpendicular Magnetic Anisotropy (PMA) is interesting in several respects: i) it allows increasing the density of memory cells on a wafer since no elliptical shape is required to stabilize the anisotropy direction, contrary to the planar systems, ii) PMA energy is usually much larger than shape anisotropy that can be obtained in planar MTJs, allowing longer memory retention at small size; iii) for a given retention time, the critical current density to write information by Spin Transfer Torque (STT) switching is strongly reduced, provided the Gilbert damping remains low enough^3^.

Perpendicular anisotropy has been shown to arise at the magnetic metal/oxide interface^4^ and can also be obtained with Co/Pt or Co/Pd multilayers. Seed and capping layers must be properly chosen since the layers texture and the elements interdiffusion play important roles in the PMA amplitude^5^,^6^. A good control of the annealing conditions is also required.

In recent years, the most commonly used structures consist of a single CoFeB storage layer and a reference layer including a Co/Pt or Co/Pd multilayer-based Synthetic AntiFerromagnet (SAF)^5^,^7^. SAF structures are composed of two magnetic layers antiferromagnetically coupled through a metallic spacer, generally Ru, thanks to RKKY coupling^8^,^9^. Usually, the SAF reference layer comprises a CoFeB layer in contact with the MgO barrier to increase the TMR value thanks to the body-centered cubic (bcc) structure that it acquires after annealing^10^. This CoFeB layer is then strongly coupled to the face-centered cubic (fcc) (CoPt) or (Co/Pd) multilayers through an ultrathin Ta layer (typically 0.3 nm thick) which ensures the structural transition from fcc to bcc and contributes absorbing boron from the CoFeB layer upon annealing and recrystallization^5^,^11^. This type of SAF structures has the advantage of providing a more stable reference with larger coercive fields than single multilayer systems. If the two magnetic layers are properly balanced, the SAF also allows drastically reducing the stray fields acting on the storage layer once the junctions are patterned into nanopillars^12^. This is a way to ensure that the two states of the memory dot can be stabilized at zero field with similar thermal stability factors. However, the use of Pt and Pd in MTJ stacks has some important drawbacks towards the manufacturability of p-MTJ stacks. Since Pt and Pd are noble metals, they are very difficult to etch by reactive ion etching techniques. They can be etched with an ion beam but this technique is not appropriate at industrial level for homogeneity reasons and because of the production of residues. Besides, these Pt- or Pd-based multilayers have a quite large total thickness and are generally made of extremely thin layers, typically 0.2–0.3 nm, which implies a very good control of the deposition parameters.

One alternative simplified stack that could overcome these drawbacks uses single CoFeB layers in both the storage and reference layers, as for example Ta/FeCoB/MgO/FeCoB/Ta^13^. However, as the difference in coercive fields between both electrodes is rather small, the stability of the reference is excessively reduced. In addition, unless etching of the top electrode is stopped at the MgO barrier, stray fields from the reference layer may be too large and may induce a loss of the dot magnetic bistability.

More recently, it was proposed to replace the standard Ta capping by a second MgO layer^14^,^15^,^16^,^17^. This second oxide interface increases significantly the perpendicular anisotropy of the CoFeB soft layer thus improving the thermal stability of the memory dots and thereby allowing using thicker storage layers with the advantage of reducing the Gilbert damping^18^. Using this type of capping layer may however reduce the TMR relative amplitude if its resistance x area (RA) product is not much smaller than the one of the main barrier. Note that even though the two barriers in^18^ are nominally identical, their RA products can be different as a result of different growth conditions (seed layers)^19^. Synthetic storage layers have also been proposed in order to enhance the thermal stability of the stored information^20^. They were however still comprising a (Co/Pd) multilayer to bring strong perpendicular anisotropy.

In this study we propose perpendicular junctions in which the reference layer is composed of two FeCoB layers antiferromagnetically coupled through a metallic spacer that is either a single Ru layer or a Ta/Ru bilayer. This reference can be located either at the bottom or at the top of the magnetic stack. The proposed structure constitutes a SAF reference not comprising any Pt- or Pd-based multilayers.

In order to extract quantitative information on these SAF structures, we will first use them as storage layers in a standard junction with a conventional bottom hard Co/Pt SAF reference. The stack is then the following: M_1_/Ru/M_2_/MgO/M_b_/(Ta)/Ru/M_t_/MgO where M_1_ is a Co/Pt multilayer, M_2_ a Co/Pt multilayer ferromagnetically coupled to a FeCoB layer through a thin (0.3 nm) Ta insertion layer and M_b_ and M_t_ are two FeCoB layers, antiferromagnetically coupled through a Ru or Ta/Ru spacer. For simplicity, in the rest of this study, the bottom Pt-based SAF will be referred to as SAF_1_ and the top Pt-free one as SAF_2_. We also use the bottom part of SAF_1_ (M_1_) as an internal reference to calibrate our VSM measurements. This allows getting information on the order of magnetization reversal as a function of applied magnetic field, and on the values of the saturation magnetizations and dead layer thicknesses. This kind of data treatment has been presented in a previous study^21^.

We then add a supplementary soft magnetic layer SL either between SAF_1_ and SAF_2_ or on top of SAF_2_, thus realizing double perpendicular junctions SAF_1_/SL/SAF_2_ or SAF_1_/SAF_2_/SL. Finally, removing the bottom Pt-based SAF_1_ allows us to realize Pt- or Pd- free perpendicular junctions: SL/SAF_2_ and SAF_2_/SL having respectively a top or bottom synthetic reference.

## Results

### Single perpendicular junctions with a synthetic storage layer

**Figure 1:**
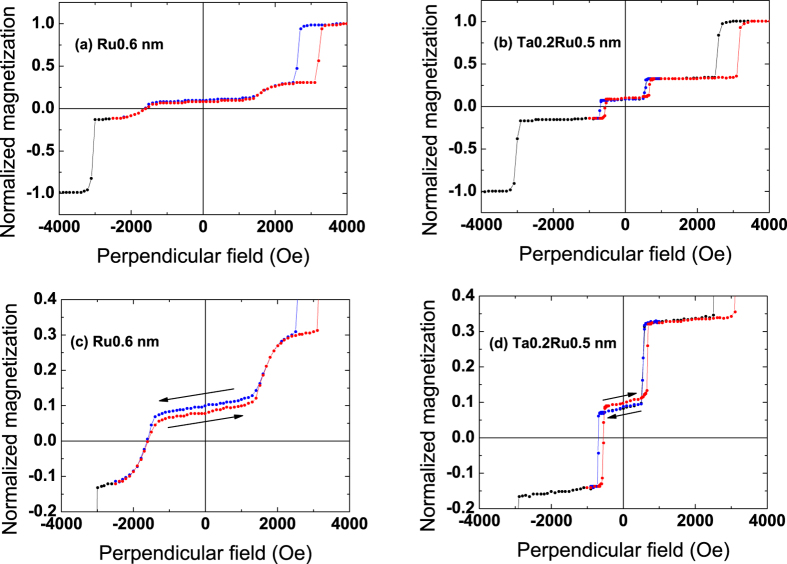
Single perpendicular SAF_1_/SAF_2_ magnetic tunnel junctions, with SAF_1_ the bottom hard reference and SAF_2_ the storage layer. Magnetic cycles measured by VSM with a perpendicular applied field. The spacer in SAF_2_ is either Ru0.6 (**a,c**) or Ta0.2Ru0.5 (**b,d**). Curves in (**c**,**d**) are zooms on the top SAF_2_.

Figure 1 presents typical VSM loops for SAF_1_/SAF_2_ junctions with a Ru0.6 (a, c) or Ta0.2/Ru0.5 (b, d) spacer in SAF_2_. The FeCoB thicknesses in SAF_2_ are fixed at 1.4 (M_b_) and 1.1 nm (M_t_), respectively. Only half the major loop, performed between 4000 and −4000 Oe, is shown (black curve). A second cycle is then performed, in which the field now decreases down to a value between −1000 and −3000 Oe, that is before the transition of SAF_1_ towards negative parallel state (blue curve) and then increases again (red curve). This allows making a major loop on SAF_2_ without modifying the bottom SAF_1_ configuration at low fields.

Coming from positive fields, the first transition around 2600 Oe corresponds to the switching of M_2_ in SAF_1_. The last transition at about −3000 Oe represents the switching of M_1_ in SAF_1_. For intermediate applied fields, the two successive transitions can be attributed to the switching of M_b_ and M_t_ that are antiferromagnetically coupled for this Ru or Ta/Ru thickness.

When looking at Fig. 1(a,b), one observes a large reduction of the coupling strength when replacing the standard Ru spacer by a Ta/Ru one. The stability of the antiparallel alignment is much smaller for the Ta/Ru spacer and ranges between ±600 Oe instead of ±1300 Oe for the Ru one. The zooms on SAF_2_ (Fig. 1(c,d)) also show that changing the nature of the spacer induces a modification of the relative positions of the antiparallel plateaus. The descending branch (in blue) of the loop is above the ascending one (in red) in the case of a Ru spacer while it is the opposite with a Ta/Ru spacer (see arrows in Fig. 1(c,d)). We have thus an under-compensated system in the first case and an over-compensated one in the other case. From another study (not detailed here), by varying the thickness of the bottom FeCoB layer, we are able to attribute the first transition of SAF_2_ at positive fields to the reversal of the bottom FeCoB layer M_b_. Knowing that M_1_, the bottom part of SAF_1_, is constant for all samples, we can use it to normalize the different magnetic contributions and compare samples. With this treatment, it appears that M_t_ remains constant while M_b_ increases by about 15% when we replace the M_b_/Ru interface by a M_b_/Ta one. This shows that magnetic dead layers are thicker for FeCoB/Ru interfaces than for FeCoB/Ta ones.

**Figure 2:**
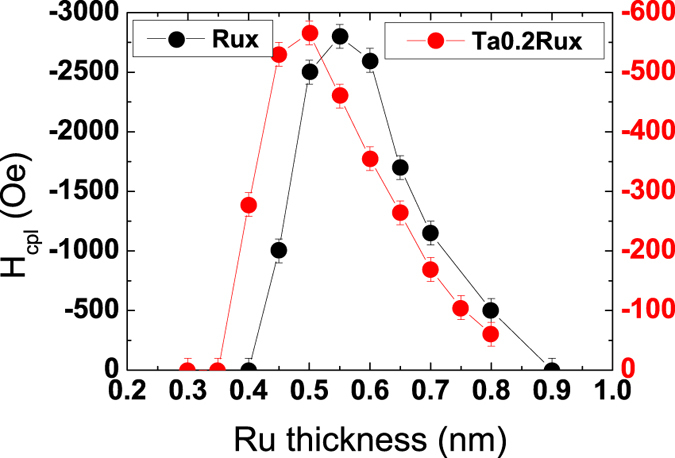
RKKY coupling as a function of Ru thickness in SAF_2_. Evolution of the RKKY coupling field as a function of Ru thickness for Rux (black dots, left axis) and Ta0.2/Rux (red dots, right axis) spacers in SAF_2_. Note the different scales for the field amplitude.

The evolution of the antiferromagnetic coupling field with a Ru or Ta0.2/Ru spacer in SAF_2_ is shown in Fig. 2 as a function of Ru thickness x. The value of this field is extracted from minor loops performed on SAF_2_ after saturating it in positive fields, decreasing the field down to 0 Oe and increasing it again. Using the usual sign convention, values of the coupling field are taken negative when coupling is antiferromagnetic. Note that in the case of ferromagnetic coupling, for small Ru thickness, we cannot extract a value for the coupling field so we arbitrarily set it at zero. The coupling field reaches a maximum around 0.5–0.6 nm and then decreases monotonously. The maximum that commonly appears around 0.9 nm in the (Pt/Co)/Ru/(Co/Pt) type of SAF structures is missing in that case. This can be explained by the fact that the RKKY coupling strongly depends on the nature of the materials at the interface with the spacer^22^. The curves are shifted towards smaller Ru thickness upon Ta insertion, but by a value (0.05 nm) smaller than that of the 0.2 nm Ta insertion. It means then that the cut-off at which ferromagnetic coupling occurs through direct coupling appears for smaller Ru thicknesses in the case of a Ta insertion. This could be due to a reduced roughness at the interface in the Ta/Ru systems. One can also note on Fig. 2 that the maximum amplitude of RKKY coupling is strongly reduced (by a factor 5) when the Ru spacer is replaced by a Ta0.2/Ru one, as already evidenced in Fig. 1. This is in good agreement with previous results indicating that the antiferromagnetic coupling is very weak in the case of a Ta spacer^15^,^23^. It is not possible to accurately determine the corresponding maximum coupling energies, since magnetic dead layers, and also probably saturation magnetizations, depend on the type of spacer and on Ru thickness. However, we can estimate them to be around 0.30 erg.cm^−2^ for a 0.6 nm Ru spacer and 0.06 erg.cm^−2^ for a Ta0.2 nm/Ru0.5 nm spacer.

**Figure 3:**
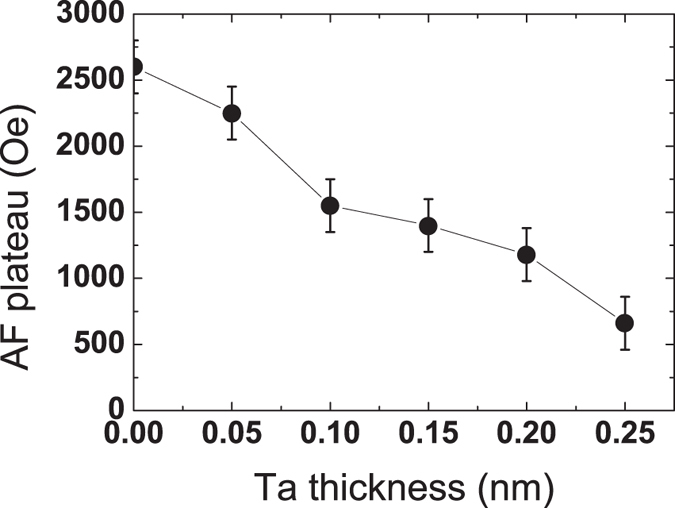
RKKY coupling as a function of Ta thickness in SAF_2_. Variation of the width of the antiparallel plateau for SAF_1_/SAF_2_ junctions as a function of Ta insertion thickness, where SAF_2_ is FeCoB1.4/Tax/Ruy/FeCoB1.1. y is adjusted to keep the maximum coupling amplitude.

To study more precisely the effect of Ta on the RKKY coupling energy, junctions with varying Ta thicknesses in the Ta/Ru spacer were prepared. In order to keep the maximum coupling amplitude, the optimal Ru thickness is adjusted using a linear approximation between Ta = 0 and Ta = 0.2 nm. The width of the antiparallel plateau as a function of Ta thickness is presented in Fig. 3. A progressive decrease of the antiferromagnetic coupling strength is observed as the Ta thickness increases. The width of the antiparallel plateau goes from about 2600 Oe without Ta insertion down to 600 Oe for a Ta thickness of 0.25 nm. This is in good agreement with our previous results in which a pure Ta spacer of 0.8 nm leads to a reduced stability range around 200 Oe^24^.

**Figure 4:**
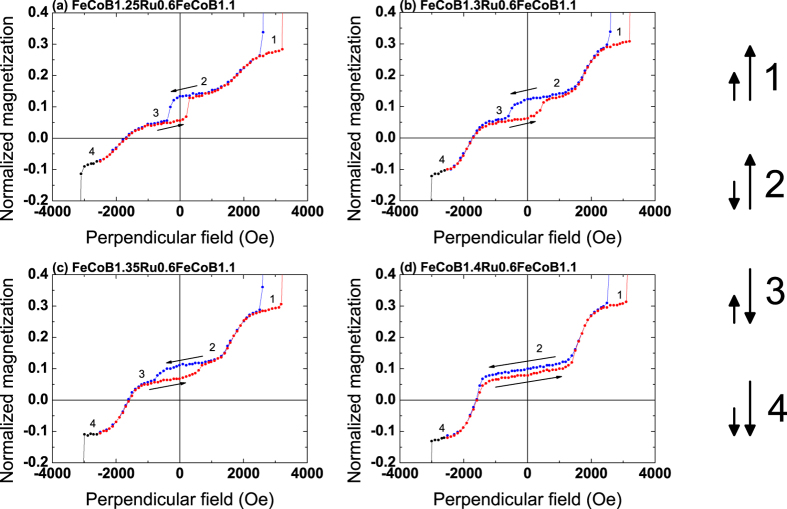
Magnetic cycles of SAF_1_/SAF_2_ structures, with SAF_1_ the bottom hard reference and SAF_2_ the storage layer. VSM measurements with a perpendicular applied field. The thickness of the bottom FeCoB layer of SAF_2_ varies between 1.25 and 1.4 nm. The y scale is chosen to focus on the magnetic loop of SAF_2_.

From the above results it appears that these SAF_2_ structures can be used as storage layers in perpendicular junctions, with an expected larger thermal stability factor Δ (Δ = KV/k_B_T, where K is the anisotropy energy, V the magnetic volume, k_B_ the Boltzmann constant and T the temperature) compared to single storage layers^20^. The field stability can be adjusted by varying either the nature or thickness of the spacer. On the contrary, if one wants to use these SAF_2_ structures as reference layers in perpendicular junctions, one should favor stackings with a single Ru0.6 spacer as they provide the largest stability plateau of the antiferromagnetic alignment. However, a strong RKKY coupling energy, compared to the perpendicular anisotropy one, may present some drawbacks. In this case, an additional transition at low fields can appear due to the switching of the net magnetization of the SAF before reaching the parallel stable state at higher fields. This phenomenon is illustrated in Fig. 4 where we show magnetic loops of samples with a Ru0.6 spacer and varying thicknesses of the bottom FeCoB layer (M_b_). Varying that thickness changes the compensation of the magnetizations of the SAF structure.

Defining J_Ru_ as the coupling energy through the Ru spacer per unit surface, H the applied field and K_effb_ the effective perpendicular anisotropy of the bottom FeCoB layer with magnetization M_b_ and thickness t_b_, the additional transition (switching from configuration 2 to configuration 3 in Fig. 4) appears when:


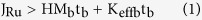


The fact that this transition does not exist in structures with Ta/Ru spacers (such as the one shown in Fig. 1(b)) is due to the much reduced RKKY coupling energy J_Ru_ in these stacks compared to the Ru0.6 spacer systems. This additional transition strongly reduces the stability range of the antiparallel plateau, and thus the stability of SAF_2_ if used as a reference layer. It occurs at about 350 Oe in the case of the thinnest bottom FeCoB thickness, as shown in Fig. 4(a). As the bottom FeCoB thickness increases, one approaches the magnetic compensation and the ascending and descending plateaus get progressively closer from one another. In the meantime, the transition between configurations 2 and 3 shifts to larger fields and disappears at the magnetic compensation (see Fig. 4(d)). This is because the torque exerted by the external field is inversely proportional to the net magnetization, and becomes less and less efficient as one approaches magnetic compensation. This transition field qualitatively varies as:


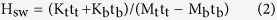


where K, t and M are anisotropy energy, effective magnetic thickness and saturation magnetization of the bottom and top FeCoB layers of SAF_2_, respectively^25^. In this macrospin approximation, Eq. 2 gives an upper limit of the experimentally measured switching field, determined by nucleation phenomena in thin films.

**Figure 5:**
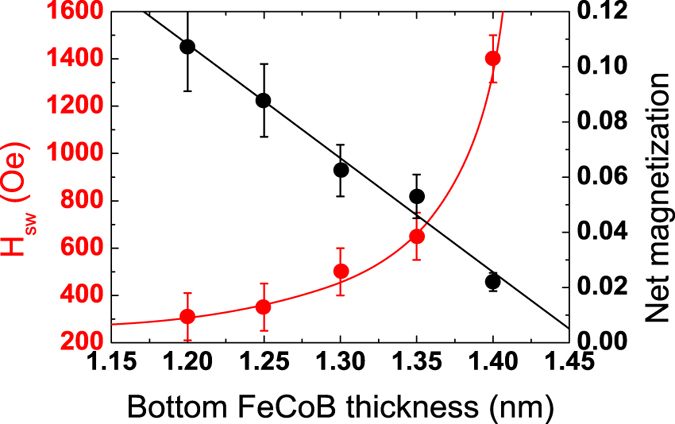
Magnetic properties of SAF_1_/SAF_2_ structures. Switching field H_sw_ of the additional transition (red dots, left axis) and net magnetization (M_t_ − M_b_)/M_1_ (black dots, right axis) as a function of the bottom FeCoB layer thickness of SAF_2_, where SAF_2_ is FeCoBx/Ru0.6/FeCoB1.1. The black line is a linear fit, while the red one is a fit to Eq. 2.

Figure 5 (left axis) shows the variation of this switching field as a function of the bottom FeCoB thickness. As for coercive fields in ferrimagnets, a divergence of the switching field is expected at the magnetic compensation point, as observed in Fig. 5 (left axis). A fit using Eq. 2 gives a compensation thickness of about 1.44 nm for the bottom FeCoB layer. Such a magnetic compensation (1.44 nm for the bottom and 1.1 nm for the top FeCoB layer) means that the bottom dead layer thickness is larger than the top one.

The variation of the normalized net magnetization of SAF_2_ (M_t_ − M_b_)/M_1_ is also plotted in Fig. 5 (right axis) as a function of the bottom FeCoB thickness. It corresponds to half the vertical opening of the low-field magnetic cycles shown in Fig. 4. Its variation with bottom FeCoB thickness (normalized to the bottom part M_1_ of SAF_1_) is expected to be linear, as observed in Fig. 5. A linear fit leads to a compensation thickness of the bottom FeCoB layer for 1.46 nm, in very good agreement with the value (1.44 nm) determined from the thickness dependence of the switching field.

Previous studies as a function of magnetic thickness (not described here) show that the top magnetic dead layer amounts to about 0.3 nm. With a mean value of 1.45 nm for the nominal bottom FeCoB thickness at the magnetic compensation (with a constant nominal top thickness of 1.1 nm), we can deduce that the bottom dead layer is about 0.65 nm thick. This leads to effective magnetic thicknesses of 0.8 nm for both magnetic layers of SAF_2_ at compensation. These thicknesses are large enough to guaranty a maximum TMR signal^21^.

These FeCoB/(Ta)/Ru/FeCoB SAF_2_ structures thus exhibit a good stability against external field. The strength of the antiferromagnetic coupling can be tuned by changing the nature of the spacer or adjusting the thicknesses of the magnetic layers. Even though the Ru0.6 spacer gives a larger stability of the antiferromagnetic configuration, it is necessary to compensate the magnetic moments on both sides of the spacer to avoid the reversal of the net magnetization (as shown in Fig. 4). This magnetic compensation within SAF_2_ should not be mistaken for the slight uncompensation often used in hard (Co/Pt)-based reference layers (here SAF_1_) to minimize stray fields in small pillars^12^. Decreasing the RKKY coupling energy by inserting a thin Ta layer avoids this additional transition, but leads to a reduced antiferromagnetic stability. From these results, it appears that such SAF_2_ structures can be used as reference layers in perpendicular junctions.

### Double perpendicular junctions

To check this hypothesis, we first investigate double perpendicular magnetic junctions, using structures studied above and introducing an additional soft layer SL either below or above SAF_2_, that is SAF_1_/SL/SAF_2_ or SAF_1_/SAF_2_/SL. Note that in the first structure considered here three MgO layers are used (two tunnel barriers and a MgO capping). This intermediate step between Pt-based (previous section) and Pt-free (next section) single junctions allows us first to keep about the same growth conditions, and second to still benefit from the possibility of normalization of the amplitude of the different magnetic transitions to the bottom part of SAF_1_, as we did above. We recently proposed such double perpendicular junctions, SAF_2_ being in this case based on Co/Pd multilayers^24^.

**Figure 6:**
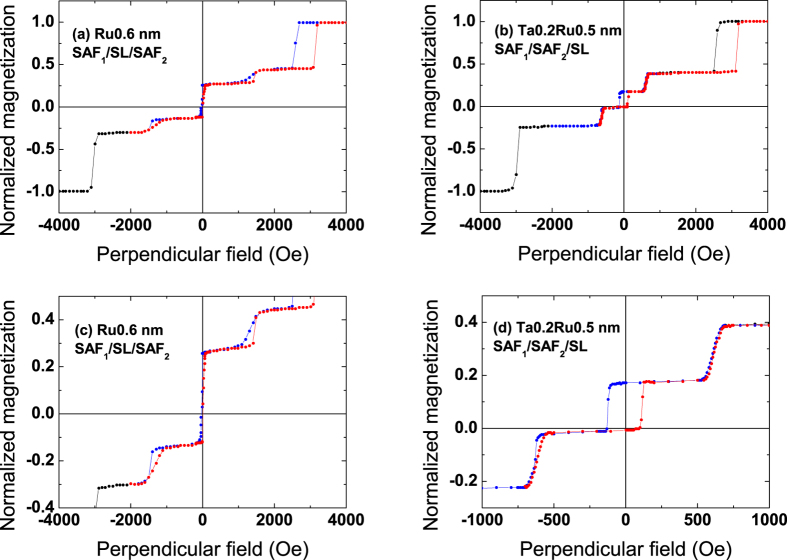
Magnetic cycles of double perpendicular junctions. Magnetic cycles measured by VSM with a perpendicular applied field for double magnetic tunnel junctions with two types of structures: (**a**,**c**) SAF_1_/SL/SAF_2_, with SAF_1_ the bottom hard reference, SL the soft storage layer, SAF_2_ the softer top reference and (**b**,**d**) SAF_1_/SAF_2_/SL, with SAF_1_ the bottom hard reference, SAF_2_ the storage layer and SL the top control layer. The bottom panel is a zoom on SAF_2_ and SL.

Figure 6(a) show the magnetic loop of a junction similar to the one presented in Fig. 1(a), in which an additional soft layer has been introduced above the first MgO barrier. This corresponds to the following double junction stack: SAF_1_/SL/SAF_2_. The soft layer SL consists in that case of two 1.2 nm thick FeCoB layers, strongly ferromagnetically coupled through a 0.3 nm Ta insertion. The additional anisotropy brought by the second MgO interface allows using in this case a large total SL magnetic thickness (2.4 nm). In the major loop, we can see all the different transitions of the layers constituting the double junction. The switching of the soft layer SL happens around 100 Oe. In Fig. 6(b) we present a junction similar to the one of Fig. 1(b), in which a soft layer is inserted above the top MgO barrier. This corresponds to the following double junction stack: SAF_1_/SAF_2_/SL. In that case, the top soft layer SL is a single FeCoB layer only 1.4 nm thick, since it is now capped with Ta instead of MgO.

The interest of these double magnetic tunnel junctions is that the STT efficiency can be strongly improved. Indeed the torques coming from the two references add up when their magnetizations are oriented antiparallel on both sides of the MgO barrier (write mode). In the case of Fig. 6(a) (with a soft storage layer in the middle of the structure), this state is obtained by starting for example from the positive saturation and applying a field of around −2000 Oe. In that case, the STT efficiency is maximal. Switching to the parallel configuration of the references (read mode) would ensure an increased stability against write error during read as the torques now subtract. The probability of inadvertently writing information with this read current is thus very small. Unfortunately, to reach this state, a field of 2000 Oe is necessary, large enough to switch again the storage layer and lead to the loss of information. The antiparallel configuration must then be kept even during the reading phase so the two MgO barriers have to be made asymmetric in order to keep a non-negligible resulting TMR signal.

The case of Fig. 6(b) (top soft layer) corresponds to a bottom reference (SAF_1_), a middle SAF storage layer (SAF_2_) and a top soft layer, usually named control layer^26^,^27^. SAF_2_ in this case is made magnetically softer by using a Ta/Ru spacer instead of a Ru one as was the case in Fig. 6(a). This structure is more efficient than the previous one, as in that case the antiparallel or parallel configurations of the reference and control layers may be obtained using a small external field (of the order of 100 Oe in Fig. 6(b)), which is large enough to switch the control layer, but insufficient to modify the information stored in SAF_2_. It is however necessary to be able to stabilize the “write” configuration of reference and control layers, that is to say make the top control layer insensitive to the STT. This may be done by introducing impurities with strong spin-orbit coupling that would lead to a larger Gilbert damping. The magnetization of the control layer could also be switched by domain wall propagation in a magnetic top stripe with perpendicular anisotropy, or by Spin-Orbit torque^28^. This last configuration leads to maximum writing efficiency and maximum stability against read current. It also allows working with identical barriers, taking full benefit of the total TMR.

Since this control layer is likely to have the smallest stability factor Δ (KV/k_B_T) of the full stack, it could be interesting to further stabilize it, by using for example a final oxide capping thanks to the additional PMA, provided by the top control layer/oxide interface. Alternatively, as suggested in ref. 27 (Fig. 13), the top control layer could be magnetically coupled to an extended magnetic stripe forming the top electrical contact to the MTJ stack (word line), its magnetization being switched by current-induced domain wall propagation in this magnetic stripe. In this configuration, the stability of the control layer would be increased thanks to its magnetic coupling to this extended magnetic stripe.

### Single perpendicular junctions with a synthetic Pt-free reference layer

The zooms in Fig. 6(b,d) show the magnetic switching of SL/SAF_2_ and SAF_2_/SL, respectively, the magnetization direction of SAF_1_ being fixed. They demonstrate that it is possible to prepare functional magnetic tunnel junctions in which the reference layer is a robust Pt- or Pd-free FeCoB/(Ta)/Ru/FeCoB SAF. Even though the stability of the Ta0.2Ru0.5 SAF is less than that of the Ru0.6 one (±550 Oe instead of ±1300 Oe), both are large enough to work with conventional FeCoB storage layers with coercive fields of less than 100 Oe.

**Figure 7:**
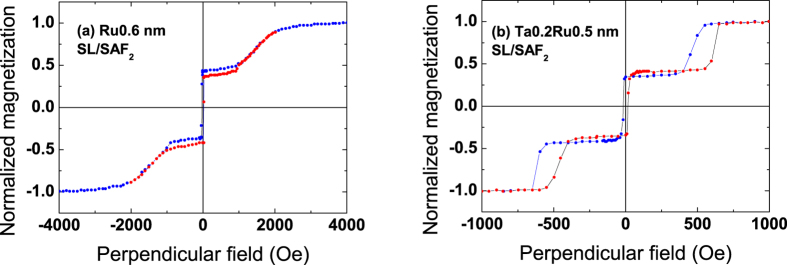
Magnetic cycles of perpendicular single junctions with a Pt-free top reference layer. Magnetic cycles measured by VSM with a perpendicular field for single junctions SL/SAF_2_ with a bottom storage layer SL = FeCoB1.2 nm and a top reference SAF_2_ that is either (**a**) FeCoB1.4/Ru0.6/FeCoB1.1 nm or (**b**) FeCoB1.4/Ta0.2/Ru0.5/FeCoB1.1 nm.

This type of single junctions is shown in Fig. 7 where the storage layer is a bottom electrode (FeCoB1.2 nm) and the top SAF reference has two different spacer layers: Ru0.6 (a) and Ta0.2Ru0.5 (b). Note that in that case, the soft FeCoB layer grows directly on a Ta spacer instead of a MgO layer as was the case in the double junction of Fig. 6(a). As we do not benefit anymore from the supplemental anisotropy brought by the bottom MgO interface, the soft layer must be thinner to maintain perpendicular anisotropy. We find again here almost the same stability ranges of the SAF as in Figs 1 and 6(a) (±600 Oe for Ta0.2/Ru0.5 and ±1000 Oe for Ru0.6).

**Figure 8:**
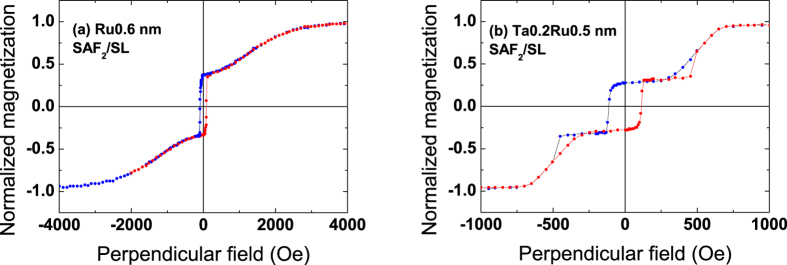
Magnetic cycles of perpendicular single junctions with a Pt-free bottom reference layer. Magnetic cycles measured by VSM with a perpendicular field for single SAF_2_/SL junctions with a top soft storage layer SL = FeCoB1.4 nm and a bottom reference that is either (**a**) FeCoB1.4/Ru0.6/FeCoB1.1 nm or (**b**) FeCoB1.4/Ta0.2/Ru0.5/FeCoB1.1 nm.

In Fig. 8, the reversed structures are presented. They are composed in that case of a bottom SAF_2_ reference with the two types of spacer ((a) Ru0.6 and (b) Ta0.2Ru0.5) and a top soft 1.4 nm thick FeCoB layer capped with Ta. Deposition is done on a thin non-magnetic layer (0.3 nm) of FeCoB covered by MgO in order to keep the same interfaces for the bottom SAF_2_ as those considered above. These junctions exhibit a slightly lower anisotropy, probably due to the difference in growth conditions, but are functional as well. Let us note that, although all structures presented here are covered with a 2 nm thick Pt layer to protect them against oxidation (see Methods), we checked that, particularly in the case of the structures shown in Figs 7 and 8 (Pt-free junctions), the Pt cap could be replaced by a Ta or Ru one without any alteration of their magnetic properties.

### Conclusion

In this study, we have presented the magnetic properties of perpendicular junctions in which the bottom reference is made of a “hard” SAF with Co/Pt multilayers (SAF_1_) and the storage layer is a “soft” SAF made of FeCoB/(Ta)/Ru/FeCoB (SAF_2_). Using the bottom layer of SAF_1_ as an internal reference, we can extract important characteristics of the two FeCoB layers of SAF_2_ such as order of switching, saturation magnetization and magnetic dead layer thicknesses. The nature of the spacer, either a single Ru layer or a Ta/Ru bilayer, strongly influences the strength of the RKKY coupling. The value of this coupling may also be tuned by changing the relative thicknesses of both FeCoB layers which modify the degree of magnetic compensation within SAF_2_. We then show that it is possible to introduce an additional soft magnetic layer (SL) at different positions in the stack to build functional double magnetic tunnel junctions, that is SAF_1_/SAF_2_/SL or SAF_1_/SL/SAF_2_. Finally, removing SAF_1_, single Pt- or Pd-free perpendicular junctions with either a top or bottom FeCoB/(Ta)/Ru/FeCoB SAF_2_ stable reference and a single FeCoB storage layer can be obtained. These structures are particularly interesting since in addition to all the advantages of a SAF reference layer in terms of reduced stray fields in patterned pillars, they allow removing Pt and Pd in the stack with all their challenges and drawbacks (material cost, tight control of very small thickness in the multilayer). Moreover, they enable drastically decreasing the total magnetic thickness of the junctions compared to standard structures with Co/Pt multilayers, which is highly beneficial to the fabrication process (etching steps in particular^29^).

## Methods

Samples in this work include six sets of tunnel junctions:

(1)Ta(3)/Pt(5)/[Co(0.5)/Pt(0.25)]*6/Co(0.5)/Ru(0.9)/[Co(0.5)/Pt(0.25)]*3/Co(0.5)/Ta(0.3)/FeCoB(1.2)/Mg(0.7)/Ox/Mg(0.5)/FeCoB(1.4)/Ta(x)/Ru(y)/FeCoB(1.1)/Mg(0.6)/Ox/Mg(0.5)/Pt(2)

Referred to as SAF_1_/SAF_2_ (Figs 1, 2, 3)

(2)Ta(3)/Pt(5)/[Co(0.5)/Pt(0.25)]*6/Co(0.5)/Ru(0.9)/[Co(0.5)/Pt(0.25)]*3/Co(0.5)/Ta(0.3)/FeCoB(1.2)/Mg(0.7)/Ox/Mg(0.5)/FeCoB(x)/Ru(0.6)/FeCoB(1.1)/Mg(0.6)/Ox/Mg(0.5)/Pt(2)

Referred to as SAF_1_/SAF_2_ (Figs 4 and 5)

(3)Ta(3)/Pt(5)/[Co(0.5)/Pt(0.25)]*6/Co(0.5)/Ru(0.9)/[Co(0.5)/Pt(0.25)]*3/Co(0.5)/Ta(0.3)/FeCoB(1.2)/Mg(0.7)/Ox/Mg(0.5)/FeCoB(1.2)/Ta(0.3)/FeCoB(1.2)/Mg(0.6)/Ox/Mg(0.5)/FeCoB(1.4)/Ru(0.6)/FeCoB(1.1)/Mg(0.6)/Ox/Mg0.5/Pt(2)

Referred to as SAF_1_/SL/SAF_2_ (Fig. 6(a,c))

(4)Ta(3)/Pt(5)/[Co(0.5)/Pt(0.25)]*6/Co(0.5)/Ru(0.9)/[Co(0.5)/Pt(0.25)]*3/Co(0.5)/Ta(0.3)/FeCoB(1.2)/Mg(0.7)/Ox/Mg(0.5)/FeCoB(1.4)/Ta(x)/Ru(y)/FeCoB(1.1)/Mg(0.6)/Ox/Mg(0.5)/FeCoB(1.4)/Ta(1)/Pt(2)

Referred to as SAF_1_/SAF_2_/SL (Fig. 6(b,d))

(5)Ta3/FeCoB(1.2)/Mg(0.7)/Ox/Mg(0.5)/FeCoB(1.4)/Ta(x)/Ru(y)/FeCoB(1.1)/Mg(0.6)/Ox/Mg(0.5)/Pt(2)

Referred to as SL/SAF_2_ (Fig. 7)

(6)Ta(3)/FeCoB(0.3)/Mg(0.7)/Ox/Mg(0.5)/FeCoB(1.4)/Ta(x)/Ru(y)/FeCoB(1.1)/Mg(0.6)/Ox/Mg(0.5)/FeCoB(1.4)/Ta(1)/Pt(2)

Referred to as SAF_2_/SL (Fig. 8)

In these structures, SAF_1_ stands for the bottom, magnetically hard Co/Pt-based synthetic layer, SAF_2_ for the magnetically softer FeCoB/(Ta)/Ru/FeCoB synthetic layer and SL for the single FeCoB or FeCoB/Ta0.3/FeCoB soft layer.

Numbers in parentheses are nominal thickness of the individual layers, in nanometer (nm). FeCoB stands for a Fe-rich Fe_72_Co_8_B_20_ amorphous alloy. All films are deposited on thermally oxidized Si wafers (500 nm SiO_2_ covered with 60 nm CuN layer) at room temperature by magnetron sputtering. The base pressure of the sputtering system is lower than 10^−6 ^Pa and working argon pressure is 0.2 Pa. Ta, Pt, Co, Ru, FeCoB, and Mg layers are dc-sputtered at a rate of about 0.05 nm/s. The first Mg layer is oxidized for 6 minutes under a dynamic O_2_ pressure of 3 Pa, and is covered by a second 0.5 nm thick Mg layer. The thickness of this first Mg layer (0.6 or 0.7 nm) is varied in order to lead to different RA products of the insulating barrier. Samples are subsequently annealed at 300 °C for 1 hour under vacuum (10^−4 ^Pa). Magnetic properties are studied using a vibrating sample magnetometer (VSM). In order to get rid of all VSM artefacts, and whenever necessary, magnetic amplitudes are normalized to that of the bottom part of the SAF_1_ reference layer, which magnetization is known and constant throughout this study.

## Additional Information

**How to cite this article**: Cuchet, L. *et al.* Perpendicular magnetic tunnel junctions with a synthetic storage or reference layer: A new route towards Pt- and Pd-free junctions. *Sci. Rep.* **6**, 21246; doi: 10.1038/srep21246 (2016).
